# Effect of long-term storage in Safe Cell+ extender on boar sperm DNA integrity and other key sperm parameters

**DOI:** 10.1186/s13028-017-0325-9

**Published:** 2017-09-11

**Authors:** Wiesław Bielas, Wojciech Niżański, Agnieszka Partyka, Anna Rząsa, Ryszard Mordak

**Affiliations:** 1 Department of Reproduction and Clinic of Farm Animals, Faculty of Veterinary Medicine, Wrocław University of Environmental and Life Sciences, pl. Grunwaldzki 49, 50-366 Wrocław, Poland; 2 Department of Immunology, Pathophysiology and Veterinary Prevention Medicine, Faculty of Veterinary Medicine, Wrocław University of Environmental and Life Sciences, ul. C. K. Norwida 31, 50-375 Wrocław, Poland; 3 Department of Internal Medicine and Clinic of Diseases of Horses, Dogs and Cats, Faculty of Veterinary Medicine, Wrocław University of Environmental and Life Sciences, pl. Grunwaldzki 47, 50-366 Wrocław, Poland

**Keywords:** Boar, Spermatozoa, DNA fragmentation, Flow cytometry, Acridine orange, SCSA, CASA

## Abstract

**Background:**

There is some controversy about the extent of changes in different sperm cell features in stored boar semen, especially regarding the potential role of the DNA fragmentation assay for assessment of sperm fertilizing ability. The aim of this study was to assess the effect of time of storage and the dynamic changes in sperm cell characteristics in normospermic boar semen stored in long-term extender, in order to determine the susceptibility to damage of particular structures of spermatozoa during cooling and storage at 17 °C for 240 h post collection. The study included five ejaculates from each of seven boars of the Polish Large White breed (n = 35 ejaculates). The sperm characteristics were assessed using a flow cytometer and a computer assisted sperm analyzer on samples at 0, 48, 96, 168 and 240 h post collection.

**Results:**

The sperm chromatin structure assay (SCSA) showed a significant abrupt increase (P < 0.01) in the DNA fragmentation index (%DFI) after 48 h of semen storage with only subtle changes thereafter, not exceeding 5% on average after 240 h of storage. The use of a combination of SYBR-14/PI stains did not reveal any significant changes in the percentage of live sperm cells up to 168 h of semen storage. A significant (P < 0.01) decrease in the percentage of live spermatozoa with intact acrosomes was observed after prolonged semen storage (168 h). A significant and progressive decrease in sperm motility was recorded during the whole period of semen storage.

**Conclusions:**

Storage of boar semen extended in long-term diluent at 17 °C for 48 h initially induced a decrease in the integrity of sperm DNA. This suggests that the structure of boar sperm DNA is susceptible to damage, especially during semen extension and at the beginning of sperm storage. These findings support the opinion that the SCSA test has only a low potential for routine assessment of boar semen preserved in the liquid state and for assessment of sperm quality changes during 10 days of semen preservation. Remarkably, the integrity of acrosomes and plasma membranes remained nearly unchanged for 7 days.

## Background

In the pig industry, the vast majority of sows are still subjected to artificial insemination (AI) with extended liquid semen, so that preservation of the fertilizing capacity of boar spermatozoa for several days remains an important target for the industry [1]. Up until now, there has been no breakthrough in the use of frozen boar semen [2], mainly due to the high sensitivity of boar spermatozoa to cooling, freezing and thawing [3]. Commonly, boar spermatozoa are stored in liquid at 15–17 °C for routine use in artificial insemination, but extenders for boar semen for storage even at lower temperatures have also been available for a number of years [4–8]. Therefore, there is a growing interest in the development of new extenders and determination of optimal storage conditions for diluted boar spermatozoa. To preserve the quality of spermatozoa in diluted boar semen during long-term storage, the choice of long-term extender is critically important [9]. Long-term extenders have certain advantages: they allow for better organization in collection centers, support long-distance transport and provide the ability to conduct research on the semen before use [4]. Unfortunately, even if extenders and lower temperatures can prolong the lifespan of spermatozoa, physiological senescence of sperm cells still cannot be completely avoided. Aging-related changes may occur, consisting of non-regulated, capacitation-like modifications [10], structural and functional changes [1], oxidative processes in cell membranes [11] and damage to DNA integrity [12]. These changes can only be partially delayed by using different extenders [13].

Among the different indicators of sperm quality during storage, motility and integrity of the sperm plasma membrane have been the most evaluated characteristics in boars. Motility is assessed by means of computer-assisted semen analysis (CASA) [14], and sperm plasma membrane integrity through flow cytometry [1, 16]. These methods are good tools for sensitive assessment of storage effects on sperm quality as well as for evaluation of new extenders and preservation methods [16]. Both sperm evaluation systems have been shown to be accurate, precise and repeatable and have greatly improved the accuracy, objectivity and reproducibility of sperm evaluation [17, 18]. However, assessment of motility and sperm membrane integrity during storage only partially addresses the lowering of sperm fertilizing potential caused primarily by aging due to free radicals. Many factors including storage length, extender type, male effect, boar age and breed affect boar sperm quality. With respect to this, assessment of the capacity of a specific extender to maintain the quality of stored boar spermatozoa should also include DNA integrity [12, 19], acrosome intactness [13, 20], mitochondrial activity [21], bacterial contamination, pH determination [9], tyrosine phosphorylation [15] and apoptotic changes [18].

One of the key features related to sperm fertility is the integrity of the nuclear DNA, whose stability largely depends on the integrity of the chromatin. Therefore, some authors recommend assessment of chromatin integrity as a good, complementary and independent indicator of sperm quality [22]. Sperm DNA fragmentation tests, such as the DNA fragmentation index (%DFI), may provide a reliable guide to identify individuals that are at risk of failing to initiate a healthy pregnancy [23]. There are several methods to assess sperm DNA fragmentation, which have been used in the assessment of boar spermatozoa. These methods are the TUNEL assay [24, 25], the sperm chromatin structure assay (SCSA) [26, 27], the Comet assay [28] and the sperm chromatin dispersion test (SCD) [29, 30].

A number of studies have indicated the potential of the SCSA for assessment of boar sperm quality [12, 26, 31] and fertility [32–34]. The negative, damaging effect of semen handling and storage on boar sperm DNA has previously been described, both with respect to liquid storage [12, 16, 28, 34, 35] and frozen storage [36–38]. Dilution conditions [28, 35, 39], time of storage of liquid semen [12] and age of boars [40], as well as variation between ejaculates within boars [12, 32, 41], may be to some extent implicated and responsible for the damage to boar sperm DNA integrity. Thus, damaged DNA is considered to be one element responsible for reduced capability of sperm cells to bind to the oviductal epithelium [41], as well as to underdevelopment of embryos [42, 43], and can lead to early embryonic or fetal death or have a dramatic impact on health of the offspring [27]. In recent studies, which investigated the relationship of flow cytometric sperm integrity assessments with boar fertility, none of the individual membrane integrity variables was significantly related to fertility except the amount of DNA damage. These studies have shown that only sperm chromatin stability had a relationship with fertility from 7 to 10 days and again from 14 to 15 days after ejaculation, dilution and long-term storage of semen [34]. Contradictory results were obtained in some recent experiments showing that the level of DNA fragmentation in liquid stored boar semen is very low for a long time [16, 19].

The aim of this study was to investigate changes in the %DFI determined by SCSA along with changes in key sperm parameters in boars to elucidate effects of boar and time of storage on sperm cell quality. During the study, semen was diluted and stored in Safe Cell+, a long-term extender for 240 h at 17 °C.

## Methods

### Animals and semen collection

Seven mature boars of the Polish Large White breed were used, ranging from 18 months to 3 years of age and selected according to the normal semen quality criteria, i.e., >50 × 10^8^ total sperm cells per ejaculate, initial motility >70%, and containing >70% morphologically normal spermatozoa. The boars in this study were routinely used in our AI center as semen donors. Boars were randomly selected among all the AI males. Thirty-five ejaculates were used in this experiment. Five ejaculates were collected from each male. Semen was collected once a week in one Polish boar station, for 5 consecutive weeks. The sperm-rich fraction of the ejaculate was collected using the gloved hand technique. Immediately after collection, the following procedures were done: initial assessment of motility with a phase-contrast microscope at 200× magnification; measurement of sperm concentration with a SpermaCue photometer Porcine (Minitüb GmbH, Tiefenbach, Germany); and preparation of smears for subsequent staining with Giemsa stain [44] and routine sperm morphology assessment (1250× magnification). The sperm-rich fraction was diluted with Safe Cell+ (IMV Technologies, l’Aigle, France) long-term extender to a final concentration of 30 × 10^6^ spermatozoa/mL to prepare conventional AI doses for fresh semen. The extended semen doses of 100 mL containing approximately 3 × 10^9^ sperm were packaged in plastic bags. They were slowly cooled down to 17 °C and subsequently transported to the Laboratory of Andrology at the Department of Reproduction, Faculty of Veterinary Medicine in Wrocław within 5 h after collection. In the laboratory, the semen doses were stored at 17 °C in a boar semen incubator (Minitüb). Samples for computer assisted sperm analysis and assessment in a flow cytometer were taken immediately after arrival at the laboratory (0 h) and again after 48, 96, 168 and 240 h of storage at 17 °C.

### Assessment of sperm cell characteristics

#### Motility

Sperm motion characteristics in extended semen were evaluated using CASA (Hamilton-Thorne Sperm Analyser IVOS version 12.2l, Hamilton Thorne Biosciences, MA, USA), under 1.89 × 10 magnification. A 3 µL aliquot of semen was placed in a Leja4 analysis chamber (Leja, Nieuw-Vannep, Netherlands) at 35 °C and evaluated. Settings of the IVOS were the following: frame acquired 45, frame rate 60 Hz, minimum cell contrast 46, minimum cell size 7, straightness threshold 45%, path velocity threshold 45 µ/s, path velocity cut off 20 µ/s, straight line velocity cutoff 5 µ/s, head size non-motile 7, head intensity non-motile 50, static head size 0.65–4.90, static head intensity 0.50–2.50, static elongation 0–87. Six fields randomly selected by a computer were analyzed for each semen sample. The motility parameters obtained by the IVOS analyzer were: VAP (average path velocity, µm/s), VSL (straight line velocity, µm/s), VCL (curvilinear line velocity, µm/s), ALH (amplitude of lateral head displacement, µm), BCF (beat cross frequency, Hz), LIN (linearity, %), MOT (total motility, %), PMOT (progressive motility, %), subpopulation of RAPID cells (velocity > mean velocity of sperm population, %). CASA was set for analysis (5 microscopic views), more than 200 spermatozoa per sample were examined.

#### Sperm membrane integrity

Sperm membrane integrity was assessed using dual fluorescent probes, SYBR-14 and propidium iodide (PI) (Live/Dead Sperm Viability Kit, Life Technologies Ltd., Carlsbad, CA, USA). Samples with a concentration of 30 × 10^6^ spermatozoa/mL were taken for the analysis. Portions (300 µL) of the samples were pipetted into cytometric tubes and 5 µL of SYBR-14 working solution was added. The working solution was obtained by diluting SYBR-14 in DMSO at a ratio of 1:49. Samples were mixed and incubated at room temperature for 10 min and then the cells were counterstained with 5 µL PI (2.4 mM working solution) for 5 min before analysis [45–47].

#### Acrosome integrity

Acrosomal damage was assessed using PNA Alexa Fluor 488 (Lectin from *Arachis hypogaea*, Merck Biosciences, Darmstadt, Germany). Ten microliter PNA Alexa Fluor 488 working solution (1 µg/mL) was added to 500 µL of sperm sample (30 × 10^6^ spermatozoa/mL) and incubated for 5 min at room temperature in the dark. Following incubation, the supernatant was removed by centrifugation (500×*g* for 3 min) and the sperm pellets were re-suspended in 500 µL of Safe Cell+. Then, 5 µL of PI (2.4 mM working solution, AO; Life Technologies Ltd.) was added to samples before cytometric analysis [48].

### Assessment of chromatin status

Sperm samples were diluted in Safe Cell+ diluent to a final concentration of 1 × 10^6^ spermatozoa/mL. The suspension (200 µL) was subjected to brief acid denaturation by mixing with 400 µL of lysis solution [Triton X-100 0.1% (v/v), NaCl 0.15 M, HCl 0.08 M, pH 1.4], held for 30 s and mixed with 1.2 mL acridine orange solution (AO; Life Technologies Ltd.) (6 µg AO/mL in a buffer: citric acid 0.1 M, Na_2_HPO_4_ 0.2 M, EDTA 1 mM, NaCl 0.15 M, pH 6). After 3 min samples were aspirated into a flow cytometer [49].

### Assessment of mitochondrial activity

The percentage of spermatozoa with functional mitochondria was estimated by combining fluorescent stains: Rhodamine 123 (R123; Life Technologies Ltd.) and PI. R123 solution (10 µL) was added to 500 µL of diluted sperm samples (50 × 10^6^ spermatozoa/mL) and incubated for 20 min at room temperature in the dark. Samples were then centrifuged at 500×*g* for 3 min and the sperm pellets were resuspended in 500 µL Safe Cell+ extender. Then PI (2.4 mM working solution) was added as previously described [48].

### Flow cytometry (FC)

Measurements were performed on a FACSCalibur (Becton–Dickinson, San Jose, CA, USA) flow cytometer. The fluorescent probes were excited by an Argon ion 488 nm laser. SYBR-14 fluorescence (cells with intact plasma membranes), PNA Alexa Fluor 488 signal (cells with damaged acrosomes), and Rhodamine 123 fluorescence (cells with active mitochondria) were detected on detector FL2. PI fluorescence (cells with damaged plasma membranes) was detected on detector FL1. Green fluorescence of acridine orange (double-stranded DNA) was detected on the FL1 detector and red fluorescence of AO (single-stranded DNA) with detector FL3. This is the standard protocol for flow cytometer analysis.

Gates were set according to forward and side scatters to eliminate particles smaller than sperm in cell aggregates. For SYBR-14/PI, PNA Alexa Fluor 488/PI and Rhodamine 123/PI fluorochrome quadrants were set on dot plots of the logs of green fluorescent events (live spermatozoa, damaged acrosomes, active mitochondria), and red fluorescent events (dead spermatozoa) and dual staining [45, 47].

The extent of DNA denaturation, expressed as the DNA fragmentation index (%DFI), was calculated based on the ratio of red/total (red + green) fluorescence for each sperm cell in the sample [22]. For each sample, two terms of DFI were evaluated: the percentage of spermatozoa outside the main population with denatured DNA (%DFI) and the percentage of spermatozoa with an abnormally high DNA stainability (%HDS). The percentage of HDS cells was calculated by setting the appropriate gate above the upper border of the main cluster of the sperm population with no detectable DNA denaturation, mainly immature cells.

Acquisitions were performed using the CellQuest 3.3 software (Becton–Dickinson, San Jose, CA, USA). The non-sperm events were gated out based on scatter properties and excluded from analysis. A total of 40 × 10^3^ events (spermatozoa) were analyzed for each sample.

### Statistical analysis

The results obtained, presented as mean ± SD of measurements on samples from 35 replicate determinations, were analyzed by ANOVA considering the time of storage and boars as the main variables. When ANOVA revealed a significant effect, values were compared by the least significant difference pairwise multiple comparison post hoc test (Tukey’s test). Differences were considered to be significant if the calculated probability of their occurring by chance was <5% (P < 0.05). The statistical model included the effect of time of storage and the interaction between boar and time of storage. All percentage data were arc sin transformed to normalize unequal variances.

The Spearman’s rank correlation coefficients were calculated to measure the statistical dependence among all variables, i.e., among all parameters assessed in the study at 0, 48, 96, 168 and 240 h of sperm storage.

## Results

### Characteristics of fresh semen

The mean volume of ejaculates collected from the boars was 264.2 ± 47.4 mL. The percentage of progressively motile spermatozoa assessed subjectively in fresh semen and the concentration of spermatozoa per mL were 76.6 ± 6.1 and 516.5 × 10^6^ ± 134.2 × 10^6^, respectively. The percentages of sperm cells with primary and secondary defects of morphology were 12.9 ± 4.6 and 3.1 ± 1.7, respectively (mean ± SD).

### CASA and flow cytometric assessment of spermatozoa

#### Motility

A gradual decrease of MOT and PMOT of spermatozoa was observed in samples stored in long-time extender (Table 1). The decrease of MOT was significant (P < 0.01) in all analysis periods, beginning from 48 h. The values for MOT of spermatozoa stored at 168 and 240 h were relatively low. Similarly, initial values of PMOT were below 25%. There were no significant differences between PMOT at 0, 48 and 96 h. A significant drop of PMOT at 168 h was noted (P < 0.001). The initial mean value of STR was 40.9% ± 4.7 and LIN was 20.4% ± 2.7 (mean ± SD). STR and LIN increased concomitantly with the decrease of ALH and PMOT. A gradual decrease in the velocity of spermatozoa was observed. A dramatic drop of VAP and the subpopulation of RAPID cells at 96 and 168 h of semen storage was observed. A significant effect on all sperm motion characteristics was shown for time of storage (P < 0.0001), and with the exception of MOT and BCF, for the factors boar and interaction of boar and time (P < 0.05, P < 0.0001) (Table 1).

**Table 1:** Motion characteristics of boar spermatozoa assessed by computer assisted semen analyzer (CASA) in semen stored for 240 h at 17 °C (mean ± SD, n = 35)

Spermatozoa	Hours of incubation					Source of variability		
	0 h	48 h	96 h	168 h	240 h	Boar	Time	Interaction
MOT (%)	77.3 ± 12.4^A^	67.3 ± 19.6^B^	55.7 ± 19.0^C^	32.0 ± 18.1^D^	20.8 ± 15.3^E^	0.0083	<0.0001	0.2397
PMOT (%)	24.0 ± 9.6^A^	21.2 ± 10.1^A^	20.2 ± 11.9^A^	11.3 ± 7.4^Ba^	6.6 ± 5.4^Bb^	0.0001	<0.0001	0.0040
VAP (µm/s)	99.0 ± 17.9^A^	98.8 ± 17.2^A^	79.1 ± 17.1^B^	65.1 ± 15.9^C^	53.2 ± 20.4^D^	0.0004	0.0000	0.0390
VSL (µm/s)	38.2 ± 6.4^A^	38.3 ± 7.4^A^	35.8 ± 8.4^AB^	32.5 ± 5.9^B^	27.7 ± 9.1^C^	0.0004	0.0000	0.0004
VCL (µm/s)	209.2 ± 37.4^A^	209.8 ± 35.7^A^	173.5 ± 37.9^B^	143.3 ± 34.3^C^	119.1 ± 47.2^D^	0.0008	0.0000	0.0399
ALH (µm)	9.4 ± 1.1^A^	9.6 ± 0.9^Aa^	9.5 ± 0.9^A^	8.9 ± 1.3^Ab^	7.7 ± 2.6^B^	<0.0001	<0.0001	0.0001
BCF (Hz)	34.8 ± 2.8^A^	34.4 ± 3.1^A^	32.8 ± 2.9^A^	33.1 ± 3.2^A^	30.4 ± 9.1^B^	0.1660	<0.0001	<0.0001
STR (%)	40.9 ± 4.7^A^	41.0 ± 4.6^A^	47.1 ± 7.0^Ba^	52.2 ± 8.4^Bb^	51.1 ± 17.4^B^	0.0001	<0.0001	<0.0001
LIN (%)	20.4 ± 2.7^Aa^	20.4 ± 2.4^Aa^	22.9 ± 3.4^AC^	25.6 ± 4.5^B^	24.6 ± 8.5^BC^	<0.0001	<0.0001	<0.0001
RAPID (%)	63.5 ± 16.4^A^	55.7 ± 21.8^A^	41.7 ± 19.6^B^	21.2 ± 15.2^Ca^	12.5 ± 10.6^Cb^	0.0039	<0.0001	0.0477

Characteristics assessed by CASA: MOT-percentage of motile spermatozoa; PMOT-percentage of progressively motile spermatozoa; VAP-average path velocity; VSL-straight line velocity; VCL-curvilinear velocity; ALH-amplitude of lateral head displacement; BCF-beat cross frequency; STR-straightness; LIN-linearity; RAPID-subpopulation of rapid cells

Different superscripts within a row indicate significant differences ^a,b^ P < 0.05; ^A,B,C,D,E^ P < 0.01

#### Viability

The percentage of spermatozoa with an intact plasma membrane (i.e., live spermatozoa) was relatively high and nearly unchanged up to 168 h of sperm storage (Table 2). At 240 h of storage a significant (P < 0.01) decrease in live cells was observed. The percentage of dead cells increased (P < 0.01) earlier, i.e., at 168 h of sperm storage. The percentage of cells that exhibited a partly green fluorescence and a partly red fluorescence (moribund, dying cells) remained unchanged during the whole storage period.

**Table 2:** Plasma membrane integrity, acrosome integrity, sperm chromatin structure assay and mitochondrial activity of boar spermatozoa in semen stored for 240 h at 17 °C (mean ± SD, n = 35)

Spermatozoa (%)	Hours of incubation					Source of variability		
	0 h	48 h	96 h	168 h	240 h	Boar	Time	Interaction
Live (SYBR+ PI−)	88.8 ± 8.2^A^	88.5 ± 6.6^A^	88.1 ± 7.7^A^	87.4 ± 7.9^A^	85.5 ± 7.7^B^	<0.0001	0.0433	0.9981
Live with intact acrosome (PNA− PI−)	82.5 ± 7.5^AB^	85.0 ± 6.1^A^	81.5 ± 9.1^AB^	80.1 ± 8.9^B^	78.6 ± 8.0^B^	<0.0001	0.0005	0.9987
%HDS	0.82 ± 0.5	0.73 ± 0.3	0.65 ± 0.3	0.79 ± 0.3	0.84 ± 0.3	<0.0001	0.1407	0.8690
%DFI	3.55 ± 2.7^A^	3.95 ± 2.5^B^	4.14 ± 2.4^BC^	4.40 ± 2.4^Ca^	4.71 ± 2.2^Cb^	<0.0001	0.0200	0.9996
Live with active mitochondria (R+ PI−)	83.0 ± 6.4	83.2 ± 6.9	82.2 ± 6.7	81.7 ± 7.2	79.1 ± 6.9	<0.0001	0.1188	0.9999

SYBR, SYBR-14; PI, propidium iodide; PNA, PNA Alexa Fluor 488 (lectin from *Arachis hypogaea*); PI, propidium iodide; %DFI (DNA fragmentation index)—the percentage of spermatozoa with DNA fragmentation; %HDS (high DNA stainability)—the percentage of spermatozoa with immature chromatin (less chromatin condensation); R, Rhodamine 123; PI, propidium iodide

Different superscripts within a row indicate significant differences ^a,b^ *P* < 0.05; ^A,B,C^ P < 0.01

#### Acrosome integrity

The percentage of live cells with an intact acrosome began to decrease significantly (P < 0.01) at 168 h of storage (Table 2). However, even on the 10th day of storage almost 80% of sperm cells possessed intact acrosomes. The percentage of live spermatozoa with damaged acrosomes remained constant during storage up to 240 h; a significant difference was detected only for the factor boar (P < 0.0001). The percentage of dead spermatozoa with intact acrosomes remained unchanged for 168 h of sperm storage. The value of this parameter increased significantly (P < 0.05) at 240 h of sperm storage whereas the percentage of sperm cells with damaged plasma membranes and damaged acrosomes remained nearly constant during the whole period of sperm storage.

#### Chromatin structure

The %DFI, describing the percentage of spermatozoa outside the main population with denatured DNA, increased significantly (P < 0.01) within a short time after semen collection and dilution, and was already apparent at 48 h of sperm storage (Table 2). At each time point of sperm assessment, a significant increase in %DFI was observed. A significant effect on sperm chromatin integrity was shown for boar and time (P < 0.0001 and P < 0.05, respectively) The percentage of spermatozoa with an abnormally high DNA stainability (%HDS), i.e., immature cells was similar at all times of sample analysis.

#### Mitochondrial activity

A gradual increase in the percentage of live spermatozoa with inactive mitochondria was observed. There was a significant difference (P < 0.05) between 0, 168 and 240 h of storage (Table 2). No significant differences were noted in the percentage of live spermatozoa with active mitochondria during storage (P > 0.05) between 0 and 240 h. There were no significant differences between the percentages of dead sperm cells with active and inactive mitochondria in consecutive measurements. However, a significant effect on these subpopulations of dead sperm cells was shown for boar and time of storage (P < 0.0001 and P < 0.05, respectively).

#### Relationships among boar sperm cell characteristics during storage

**Fig. 1:**
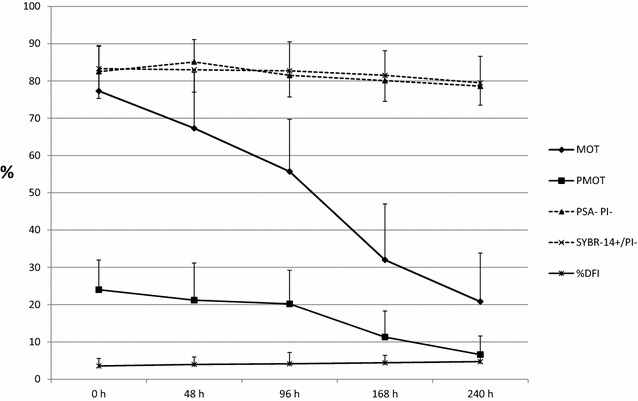
Changes in motility, progressive motility, acrosome integrity, plasma membrane integrity and DNA fragmentation in spermatozoa stored for 240 h at 17 °C (mean ± SD, n = 35). Characteristics assessed by computer assisted sperm analyzer: MOT-percentage of motile spermatozoa; PMOT-percentage of progressively motile spermatozoa. Characteristics assessed by flow cytometer: PNA− PI− live spermatozoa with intact acrosome; SYBR-14+/PI− live spermatozoa; %DFI the percentage of spermatozoa with DNA fragmentation

In Fig. 1, the relationship between the %DFI and motility (MOT and PMOT) and the viability, acrosome and plasma membrane integrity characteristics, is shown for 35 ejaculates, representing different patterns of changes in these boar sperm cell parameters during storage.

**Fig. 2:**
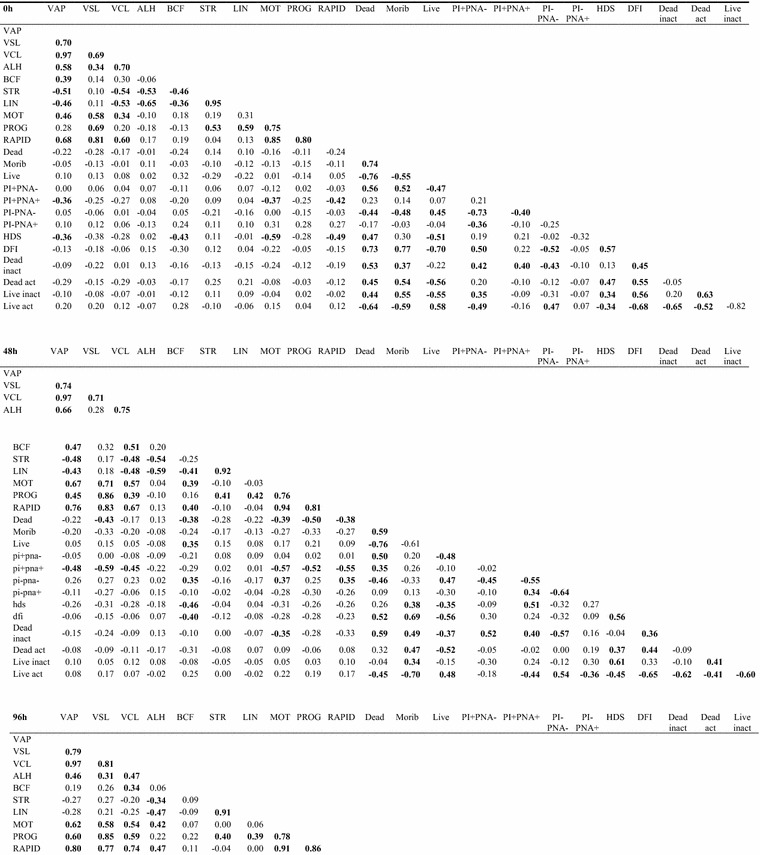
Spearman’s correlation coefficients between all analyzed sperm parameters in boar spermatozoa stored for 240 h at 17 °C (n = 35). Values in bold font—statistically significant correlation (P < 0.05). VAP-average path velocity; VSL-straight line velocity; VCL-curvilinear velocity; ALH-amplitude of lateral head displacement; BCF-beat cross frequency; STR-straightness; LIN-linearity; mot-percentage of motile spermatozoa; PMOT-percentage of progressively motile spermatozoa; RAPID-subpopulation of rapid cells; Live—SYBR+ PI; PI− PNA− live with intact acrosome; HDS—%HDS (high DNA stainability) the percentage of spermatozoa with immature chromatin; DFI—%DFI (DNA fragmentation index) percentage of spermatozoa with DNA fragmentation; Live act—live with active mitochondria (Rhodamine+ PI−)

**Fig. 3:**
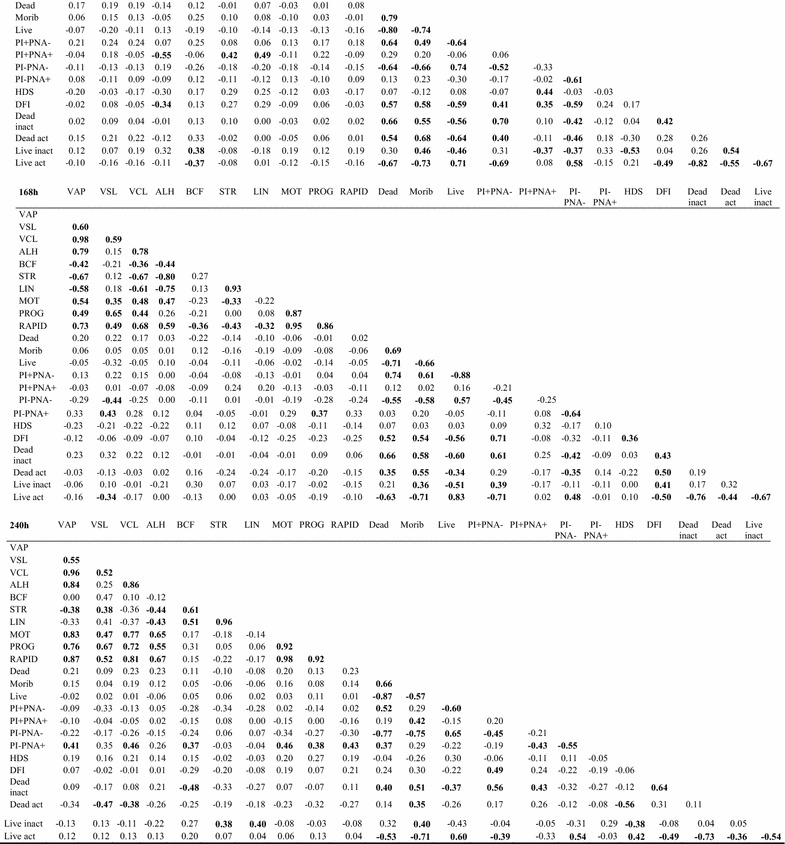
Spearman’s correlation coefficients between all analyzed sperm parameters in boar spermatozoa stored for 240 h at 17 °C (n = 35). Values in bold font—statistically significant correlation (P < 0.05). VAP-average path velocity; VSL-straight line velocity; VCL-curvilinear velocity; ALH-amplitude of lateral head displacement; BCF-beat cross frequency; STR-straightness; LIN-linearity; mot-percentage of motile spermatozoa; PMOT-percentage of progressively motile spermatozoa; RAPID-subpopulation of rapid cells; Live—SYBR+ PI; PI− PNA− live with intact acrosome; HDS—%HDS (high DNA stainability) the percentage of spermatozoa with immature chromatin; DFI—%DFI (DNA fragmentation index) percentage of spermatozoa with DNA fragmentation; Live act—live with active mitochondria (Rhodamine+ PI−)

Statistically significant correlations between motility parameters at consecutive hours of semen incubation (Figs. 2, 3) were observed. The experiment also revealed many statistically significant correlations among the majority of the structural parameters of boar sperm cells during incubation. Moreover, the %DFI and %HDS were well correlated with structural parameters at 0 h of incubation. After that only %DFI was strongly correlated with sperm structural features while %HDS lost that feature. At 240 h of incubation, neither parameter presented significant correlations with structural characteristics of sperm cells. Overall, there were no strong correlations between motility parameters and structural parameters of boar spermatozoa stored in the liquid state.

## Discussion

Artificial insemination in pigs is mostly done using boar semen preserved in the liquid state at 16–17 °C. Therefore, semen in the present study was diluted and stored in extender at 17 °C. While extenders for boar semen for storage at lower temperatures have been available for a number of years [4–8], these are not sufficiently effective for practical use in pig AI and further work is needed to produce efficient low temperature extenders.

The decrease of MOT and PMOT is similar to values obtained by others [18, 50]. The percentages of motile sperm stored for 5 days in long-term extenders X-cell, Androstar and Mulberry III were 55.6, 49.9 and 80.5, respectively [18].

We found that the percentages of boar spermatozoa with intact plasma membranes were usually more that 80% and did not change significantly during storage for 168 h. Similar results concerning sperm plasma membrane integrity in stored boar spermatozoa were obtained by others [18].

The study revealed a relative stability of sperm acrosomal membranes during storage of boar semen in the liquid state. It should be noted that there was a discrepancy between apparent loss of sperm motility and unchanged percentages of acrosome intact spermatozoa at consecutive measurements up to 168 h of semen storage.

Based on a total of 35 ejaculates collected from seven boars over a period of 5 weeks, 17.1% of the sperm samples showed >3%DFI, 11.4% showed >5%DFI, and 5.7% showed >10% of %DFI. Thus, our findings confirm previous observations about relatively low levels of %DFI in fresh boar semen [16, 26, 31, 34].

It has been proved that differences in sperm DNA damage between ejaculates can result from external factors such as collection procedure, handling, dilution or internal factors, e.g., inherent chromatin packaging of the spermatozoa, the composition of seminal plasma and of accessory gland fluid including zinc ions, zinc-binding proteins, low molecular weight antioxidants and proteins with antiperoxidant properties [16, 34]. There is a current debate about whether these intrinsic or extrinsic factors cause different reactions of the sperm chromatin to the SCSA procedure. In our study, the influences of external factors were minimized just as in earlier studies [16, 34]. However, in the present study, sperm rich fractions were used, which may mean that the antioxidant properties of the entire seminal plasma were reduced [51].

In the present study, the average %DFI of spermatozoa in fresh semen after extension (0 h) in 35 ejaculates from seven boars was 3.55%. We found an initial significant, abrupt rise of %DFI at 0 h and again at 48 h of incubation in long-term extender. The %DFI obtained here increased slightly but significantly during the 240 h of storage after collection, up to an average of 4.71 ± 2.2%. This increase was greatest (+34.48%) between day 0 and day 2, and in most boars the percentages of spermatozoa with fragmented DNA were almost always lower than 5% up to 240 h of storage. Our data agree with the results of Broekhuijse et al. [34] who reported that the %DFI at day 0 was 3.15% and increased to 4.19% during 15 days of liquid storage, and the greatest increase of %DFI was observed between day 0 and day 1.

It was also previously reported that extended boar spermatozoa showed an increase in DNA instability from day 0 to day 4 in some extenders [12, 28]. Contrary to this, De Ambrogi et al. [19] suggested that the customary storage of boar semen for 96 h at 17 °C was too short an interval to cause loss of integrity in nuclear DNA. Similar results were obtained by Waberski et al. [16] in the first part of their study. However, in the second part of their study, they obtained a slight but significant increase in mean %DFI results from 2.2% initially up to 2.7% at 120 h of semen storage.

In our study, only two out of 35 ejaculates showed an increase of %DFI above 10% (12.6; 10.8) during 240 h of storage. This is in accordance with findings of other authors who showed a significant increase of DFI during 168 h of storage in only three out of 42 ejaculates [16].

The results support the concept of relatively low sensitivity of boar sperm DNA to defragmentation during storage of liquid semen [12, 16, 19, 34]. The differences in DNA fragmentation on different days of storage were generally rather low compared to changes in motility or sperm viability. It can be assumed that these slight differences may have no biological significance. This assumption supports the conclusion presented by Hernandez et al. [38] who stated that the low overall DNA damage observed in frozen-thawed spermatozoa seemed to have little biological importance. Waberski et al. [16] also demonstrated that evaluation of sperm chromatin structural integrity by the SCSA has only limited value for identifying deficiencies in normospermic fresh or stored boar spermatozoa.

The values of %HDS in boars of the Polish White Large breed were similar to values obtained by others [12] for Landrace, Danish Large White and Hampshire boars. We did not observe any trend of decrease or increase of %HDS during sperm storage. The similarity of HDS values during the whole period of semen storage is probably due to the fact that the %HDS value is mainly determined by the initial integrity of DNA structure resulting from the quality of spermatogenesis. In humans, the population of %HDS is supposedly composed of immature cells that lack chromatin condensation [52] and may also represent doublets or triplets of spermatozoa. This is consistent with our observation that storage of boar semen did not increase the %HDS population. Thus, it seems that %HDS is not a useful marker of changes in the quality of liquid stored boar spermatozoa.

Significant differences in chromatin structure of stored spermatozoa between individual boars were also detected. The study included only seven boars, but there were differences among males with respect to the quality of their sperm characteristics during storage. We discovered a significant influence of boar on chromatin integrity (Table 2) in spermatozoa stored for 240 h at 17 °C, and also on the sperm motion characteristics (with the exception of BCF) (Table 1), on plasma membrane integrity (Table 2), on acrosome integrity (Table 2) and on mitochondrial activity (with the exception of dead sperm cells with active mitochondria) (Table 2). These study reveal that there is individual variation among boars concerning preservation of DNA integrity during storage, which is in accordance with Fraser and Strzeżek [25] and Sutkeviciene et al. [53]. This indicates that the effect of individual boar is of great importance concerning sperm quality during longtime storage and must always be taken into account in the assessment both of DNA fragmentation and other sperm variables. No previous studies have investigated sperm DNA integrity using the SCSA parameters in the semen of normospermic, healthy boars of the Polish Large White breed. However, our preliminary results need to be investigated further in a larger study to evaluate and understand the precise mechanism maintaining sperm DNA integrity.

Sperm characteristics, especially all motility and structural parameters, were also significantly affected by the storage time. This means that the time points when these characteristics were assessed during storage had a significant impact on these properties of the stored boar sperm cells.

The interaction of boar and time of incubation as a source of variability only affected motility parameters (without MOT) assessed by CASA during storage, which indicates that the boar factor as well as time of semen storage play important roles in assessment of these motility traits during long-term liquid storage of boar semen. Meanwhile, the non-significant interactions between boar and time points in the remaining assays of sperm characteristics indicate that influences of boar and time of semen storage were homogeneously distributed among the sperm parameters studied. This may mean that these characteristics of spermatozoa provide an additive value in assessing the quality of long-term liquid-stored boar spermatozoa.

Significant negative correlations among SCSA variables and most classical sperm quality parameters in fresh and cryopreserved semen were shown in rams [54], bulls [55] and humans [22]. The significant correlations between SCSA and classical sperm quality parameters suggest that, taken together, both types of assays are better predictors of sperm quality and male fertility than each one separately. The negative correlation between %DFI and sperm viability was also detected in stored boar spermatozoa [12]. In another study [19] when ejaculates from only four boars were included, the increase of %DFI was accompanied by increased deterioration of sperm plasma membrane integrity during storage. Other researchers [34] did not find a significant correlation between the %DFI and the standard boar sperm variables during long-term storage. We found significant correlations between %DFI and %HDS and structural sperm parameters at 0 h of storage after which the only significant correlations were observed for %DFI. It may be concluded that %DFI changes are associated with the disruption of other sperm structures during storage. On the other hand, %HDS is the parameter associated with abnormalities of spermatogenesis and is only partly independent from storage time. Correlations between both parameters and structural features were lost at 240 h of storage. This may indicate that assessing DNA integrity has an additive value for standard sperm assessment only in cases of extremely long storage times.

It is noteworthy that detailed analysis of correlations between values obtained in the present study revealed high, significant correlations among the majority of motility parameters. Therefore, it may be suggested that routine analysis on 1–2 motility parameters is adequate for proper evaluation of motion properties of stored boar spermatozoa. It should be borne in mind that we revealed almost no correlation between motility features and structural sperm parameters. The high correlation between all motility parameters and the lack of correlation between motility and structural features was characteristic during the whole time of sperm storage. Therefore, motility and structure may be treated as separate, partly independent features that always have to be separately assessed.

We did not observe abrupt changes of mitochondrial activity over time in the populations of live and dead spermatozoa. It is obvious, that in the population of dead spermatozoa the proportion of cells with inactive mitochondria remained nearly unchanged at consecutive assessment points. However, it is more difficult to understand why changes in mitochondrial potential were so subtle in the group of live cells in spite of the rapid decrease in progressively motile spermatozoa. It appears that the dynamics of the increase in percentages of live cells with inactive mitochondria were similar to the dynamics of other tests performed on the flow cytometer rather than the dynamics of motility changes recorded on CASA.

## Conclusions

The most sensitive method for assessing changes in sperm cell features during storage at 17 °C are those describing populations of motile cells and parameters related to speed of motility. Plasma and acrosome membrane integrity and mitochondrial function characteristics are relatively resistant to storage in long-term semen extender and change to a lesser degree. Although increased DNA fragmentation was revealed, the extent of these changes was relatively low and it appears that extenders efficiently protect DNA structure. These findings support the opinion that the SCSA test has relatively little value for routine evaluation of changes in boar sperm characteristics during semen storage in long-term extender.
